# A Mechanism Underlying Attenuation of Recombinant Influenza A Viruses Carrying Reporter Genes

**DOI:** 10.3390/v10120679

**Published:** 2018-11-30

**Authors:** Xiujuan Zhao, Lin Wang, Qinghua Cui, Ping Li, Yanyan Wang, Yingying Zhang, Yong Yang, Lijun Rong, Ruikun Du

**Affiliations:** 1 College of Pharmacy, Shandong University of Traditional Chinese Medicine, Jinan 250355, China; xiujuan95@163.com (X.Z.); lynn942@163.com (L.W.); user753951@163.com (Q.C.); liping9309@163.com (P.L.); yyw0196@163.com (Y.W.); 2 Shandong Provincial Collaborative Innovation Center for Antiviral Traditional Chinese Medicine, Jinan 250355, China; zyy8965@163.com (Y.Z.); yy7204@163.com (Y.Y.); 3 College of Traditional Chinese Medicine, Shandong University of Traditional Chinese Medicine, Jinan 250355, China; 4 Scientific Research Centre, College of Medicine, Shandong University of Traditional Chinese Medicine, Jinan 250355, China; 5 Department of Microbiology and Immunology, College of Medicine, University of Illinois at Chicago, Chicago, IL 60612, USA

**Keywords:** influenza A virus, reporter virus, attenuation, mechanism, noninfectious particles, vRNA replication

## Abstract

Influenza A viruses (IAV) carrying reporter genes provide a powerful tool to study viral infection and pathogenesis in vivo, however, incorporating a non-essential gene into the IAV genome often results in virus attenuation and genetic instability. Very few studies have systematically compared different reporter IAVs, and most optimization attempts seem to lack authentic directions. In this study, we evaluated the ratio of genome copies to the number of infectious unit of two reporter IAVs, PR8-NS1-Gluc and PR8-PB2-Gluc. As a result, PR8-NS1-Gluc and PR8-PB2-Gluc produced 41.4 and 3.8 genomes containing noninfectious particles respectively for every such particle produced by parental PR8 virus. RdRp assay demonstrated that modification of segment NS by inserting reporter genes can interfere with the replication competitive property of the corresponding vRNAs, and the balance of the 8 segments of the reporter IAVs were drastically impaired in infected cells. As a consequence, large amounts of NS-null noninfectious particles were produced during the PR8-NS1-Gluc packaging. In summary, we unravel a mechanism underlying attenuation of reporter IAVs, which suggests a new approach to restore infectivity and virulence by introducing extra mutations compensating for the impaired replication property of corresponding segments.

## 1. Introduction

Influenza A virus (IAV) is a major cause of respiratory illness in humans, accounting for up to 500,000 annual deaths worldwide [1]. IAV belongs to the Orthomyxoviridae family of enveloped viruses, and its genome contain 8 single-stranded, negative-sense RNA segments (vRNAs), coding for at least 11 proteins [2]. The segments vary in length from 2341 to 891 nucleotides (nts) and are named after the main proteins they encode, including PB2 (Polymerase basic 2), PB1 (Polymerase basic 1), PA (Polymerase acid), HA (Hemagglutinin), NP (Nucleoprotein), NA (Neuraminidase), M (Matrix) and NS (Non-structural) [2]. The vRNAs are packaged as viral ribonucleoproteins (vRNPs) containing multiple copies of NP and a RNA dependent RNA polymerase (RdRp), which consist of one subunit each of PA, PB1 and PB2 [3]. The central coding region of the vRNAs is in antisense orientation and flanked at both terminal ends by non-coding regions (NCRs) [4]. Although the NCRs differ in length and in sequence between vRNAs, the first 13 nts at the 5’ end and the first 12 nts at the 3’ end are highly conserved, partially complementary and segment-independent [5], imposing a panhandle structure which is critical for initiation of transcription and replication [6].

Upon virus entry into host cells, the vRNPs are translocated to the nucleus, where viral genome replication and gene transcription take place. During these processes two different classes of (+)RNAs, the complementary RNA (cRNA) and viral messenger RNA (vmRNA), are synthesized. The cRNAs act as templates for producing new vRNAs, while the vmRNAs are translated for viral proteins [7].

In the past two decades, the reverse genetics of IAV was extensively developed and allows manipulation of viral genomes in laboratory, generating recombinant viruses [8,9]. One application of this technique is to introduce mutations into the virus genome to study the impact of those mutations on virus biology, e.g., IAV reverse genetic systems were used to define nucleotides that are critical for vRNAs packaging [10,11]. Also a common application is construction of chimeric viruses to identify pathogenic determinates or develop novel live attenuated vaccines [12]. For example, live attenuated IAVs could be achieved by truncation of NS1 [13], deoptimization of genetic codes [14,15], or rearrangement of virus genome [16,17]. In addition, a reassortment-incompetent live influenza A virus was generated using an attenuated influenza B virus expressing the ectodomain of the influenza A hemagglutinin protein [18,19]. Recently, IAVs expressing reporter proteins have provided a powerful tool to investigate virus biology in vivo [20,21]. Moreover, reporter viruses have tremendous potential in antiviral screening, and the visualization of infection in animal models [22,23,24].

Unfortunately, incorporation of a non-essential gene into the IAV genome often results in virus attenuation and genetic instability. A recombinant virus NS1-GFP, which was the first replication-competent reporter IAV, is attenuated both in vitro and in vivo, and shown to lose reporter activity after passage in cell culture and mice [25]. Great efforts have been made to mitigate this problem. Heaton et al. generated a reporter IAV by inserting a luciferase reporter protein in a new site [24]; Tran et al. used a very small but extremely bright luciferase variant NanoLuc as the reporter [26]; Reuther et al. generated a variety of stable reporter IAVs by genetic engineering of the NS gene segment with a second PTV-1 2A peptide [27]. Most recently, Cai et al. used directed evolution of a luciferase-expressing IAV in mice to restore replication kinetics and virulence, increase the bioluminescence signal, and maintain reporter gene expression [28]. However, the mechanism underlying the attenuation is still not clear, and challenges in the development and application of reporter-expressing IAVs remain.

Previously, we generated a replication-competent recombinant IAV carrying *Gaussia* luciferase (Gluc) PR8-NS1-Gluc, which shows a delay in replication kinetics and >1 log reduction of the virus titer [29]. In this study, we investigated the mechanism for attenuation of PR8-NS1-Gluc, and compared it to another reporter IAV PR8-Gluc (referred to as PR8-PB2-Gluc here) which was constructed using an updated strategy [24]. Our results indicate that modification of NS segment by insertion of Gluc gene greatly impairs its vRNA replication efficacy, consequently interfering with its packaging into progeny virions and producing a large amount of NS-null noninfectious particles. Our study reveals a mechanism by which the reporter IAVs attenuate, providing a feasible approach for further optimization.

## 2. Materials and Methods

### 2.1. Cell Lines and Viruses

Human embryonic kidney 293T cells (HEK293T) and Madin-Darby canine kidney (MDCK) epithelial cells were purchased from ATCC (American Type Culture Collection, Manassas, VA, USA) and grown in Dulbecco’s modified Eagle’s medium (DMEM; Cellgro, Herndon, VA, USA) supplemented with 10% fetal bovine serum (FBS; Gibco, Waltham, MA, USA), 1000 units/mL penicillin and 100 μg/mL of streptomycin (Invitrogen, Carlsbad, CA, USA). Infections were performed in Opti-MEM containing 2 μg/mL *N*-tosyl-l-phenylalanine chloromethyl ketone (TPCK)–trypsin (Sigma-Aldrich, St. Louis, MO, USA). All cells were cultured at 37 °C in 5% CO_2_.

Reporter virus PR8-NS1-Gluc and parental virus PR8 were generated and stocked as previously described [29]. Reporter virus PR8-PB2-Gluc was rescued with pDZ-PB2-Gluc along with the other seven IAV rescue plasmids pDZ-PA, -PB1, -NP, -HA, -NA, -M, and -NS1 as previously described [29]. The plasmid pDZ-PB2-Gluc, pPolI-NS and pPolI-NS-Gluc were kindly provided by Dr. Balaji Manicassamy (the University of Iowa). The TCID50 values of viral stocks were determined by inoculation of serial 10-fold dilutions of stock virus onto MDCK cells, and the titer was calculated by the Reed–Muench method.

### 2.2. Single-Cycle and Multiple-Cycle Replication Assay

In order to perform a virus replication assay, MDCK cells grown in 6-well plates were infected by indicated viruses at multiplicities of infection (MOI) of 1 or 0.01 TCID_50_ per cell respectively for single-cycle or multiple-cycle replication assay. After 1 h incubation at 37 °C, the virus was removed and the cells were washed, followed by adding fresh Opti-MEM containing 2 µg/mL TPCK–trypsin. Aliquots were removed at various time points and titrated by determining the TCID_50_ values.

### 2.3. qPCR Analysis of Packaged vRNAs

MDCK cell derived stocks of indicated viruses were clarified by centrifugation at 8000× rpm for 10 min, and passed through a 0.45 μm filter. The vRNAs of virus stocks were extracted using Simply P Virus RNA Extraction Kit (Bioflux, Zhejiang, China) and reverse transcribed using a universal 3’ primer 5’-GATCGCTCTTCTGGGAGCRAAAGCAGG-3’ and PrimeScript RT reagent Kit (Takara, Beijing, China) according to the manufacturers’ instructions. The RT product were then used as templates for quantitative PCR (qPCR) with segment specific primers (Table 1) separately.

To determine the concentration of segment M, plasmid pDZ-M was serial log diluted and used to generate a standard curve range from 1 × 10^8^ to 100 copies. The standard curve is the linear regression line through the data points on a plot of Ct (threshold cycle) versus logarithm of standard sample concentration.

In order to examine the composition of the 8 vRNA segments of indicated viruses, the relative concentrations of vRNAs were normalized to that of parental PR8 and presented as the ratios of each vRNA segments versus M. The standard deviations were calculated based on three replicates.

### 2.4. Cell Based RdRp Assay

Cell based influenza RdRp assay was modified as previously described. Briefly, 293T cells growing in 6 well plates were cotransfected with plasmid pDZ-M (200 ng), expression plasmid constructs pFluNP (480 ng), pFluPB1(240 ng), pFluPB2 (240 ng), pFluPA (240 ng), for NP, PB1, PB2 and PA expressions, as well as equimolar amounts of pPolI-NS (185 ng) and pPolI-NS-Gluc (225 ng) respectively. At 36 h post transfection, the transfected cells were harvested, and total RNAs were extracted using Simply P Total RNA Extraction Kit (Bioflux, Zhejiang, China).

To detect the replication efficacy of vRNAs of NS and M segments, reverse transcription was performed using the 3’ primer 5’-GATCGCTCTTCTGGGAGCAAAAGCAGG-3’ and PrimeScript RT reagent Kit (Takara, Beijing, China). RT product was then analyzed by qPCR as described above and the ratios of vRNA NS versus M were presented.

To examine the mRNA level of NS and M gene, the extracted RNAs were transcripted using oligo(dT) as the primer, and qPCR were performed using primers specific to NS, M and NP respectively.

### 2.5. Analysis of vRNA Replication in Infected Cells

MDCK cells were infected with indicated viruses at an MOI of 1 TCID_50_/cell. After incubation for 1 h at 37 °C, the cells were washed and fresh Opti-MEM containing 2 μg/mL TPCK–trypsin were added. Infected cells were harvested at indicated time points, and total RNAs were extracted using Simply P Total RNA Extraction Kit (Bioflux, Zhejiang, China). The vRNAs were transcripted and analyzed by qPCR as described above. The copies of M-segment vRNA derived from 50 ng total RNA were calculated. The formula 2^−ΔΔCt(X,Y,T)^ = 2^−((Ct[X,Y,T]−Ct[X,M,T])−(Ct[PR8,Y,T)−Ct[PR8,M,T]))^ was used to calculate the relative concentration of vRNA Y of virus X compared to parental PR8 at indicated time point (T), which was presented as the ratios of each vRNA segment versus M. The relative concentration kinetics were further analyzed using the formula Con(X,Y,T) = 2^-ΔΔCt(X,Y,T)^ × (Con[PR8,M,T]/Con[PR8,M,T_0_]), where T_0_ represent 0 h post infection. The standard deviations were calculated based on three replicates.

## 3. Results

### 3.1. Comparison of the Replication Kinetics of Two Reporter IAVs PR8-NS1-Gluc and PR8-PB2-Gluc

**Figure 1:**
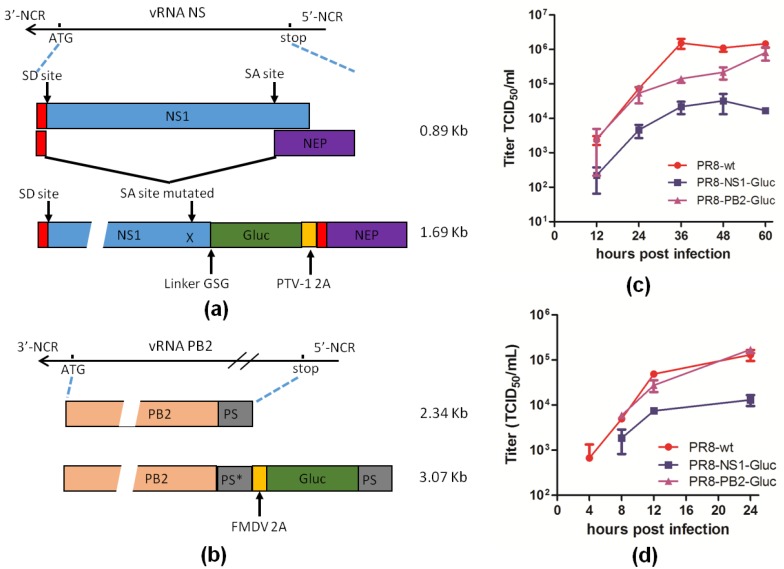
Comparison of two reporter IAVs, PR8-NS1-Gluc and PR8-PB2-Gluc. Schematic representation of the NS (**a**) and PB2 (**b**) segment encoding the GLuc reporter gene. SD/SA—Splice donor/acceptor sites. PS* represents silent mutations of the original packaging signal; PS represents the duplicated original packaging sequence. (**c**) Multiple-cycle growth curves of indicated IAVs. MDCK cells were infected with indicated viruses at an MOI of 0.01 and incubated for the indicated time points for titration. The standard deviations were calculated based on three independent experiments. (**d**) Single-cycle growth curves of indicated IAVs. MDCK cells were infected with indicated viruses at an MOI of 1 and incubated for the indicated time points for titration. The standard deviations were calculated based on three independent experiments.

Reporter IAVs PR8-NS1-Gluc and PR8-PB2-Gluc were generated by two different strategies. PR8-NS1-Gluc was constructed by fusion of the *Gaussia* luciferase gene to NS1 (Figure 1a) [29], while PR8-PB2-Gluc was modified by insertion the same reporter gene into PB2, the longest segment of IAV genome, which is believed to have higher tolerance with exogenous sequences (Figure 1b) [24]. Our previous data demonstrated that reporter IAV PR8-NS1-Gluc showed a delay in replication kinetics and >1 log reduction in virus titer when grown in MDCK cells [29], while PR8-PB2-Gluc showed about 1 log reduction in titer when grown in eggs, compared to the parental PR8 [24]. To better understand the difference in replication properties of the two reporter IAVs, both the multiple-cycle and single-cycle replication assays were performed in MDCK cells.

As shown in Figure 1c, for multiple-cycle replication assays, PR8-NS1-Gluc showed a delay in replication kinetics at as early as 12 h (h) post infection (p.i.), while PR8-PB2-Gluc did not show any delay until 24 h p.i. At 36 h p.i., titers of both PR8 and PR8-NS1-Gluc peaked, with PR8-NS1-Gluc having a 1.8 log reduction compared to PR8. Although PR8-PB2-Gluc also showed a 1 log reduction at 36 h p.i., it kept growing till 60 h p.i., and the titer almost reached the highest titer of PR8 eventually.

Similar results were observed for single-cycle replication assays. PR8-NS1-Gluc showed significant reduction in titers all through the infection, compared to parental PR8, While PR8-PB2-Gluc displayed nearly identical kinetics as PR8 (Figure 1d).

These results suggest that both reporter IAVs are attenuated in vitro, however, PR8-PB2-Gluc is less attenuated.

### 3.2. The Genome Copies-to- TCID_50_ Ratio of PR8-NS1-Gluc is Greatly Increased

The IAV genome consists of 8 segments, which complicates the genome packaging of progeny virions. Since PR8-NS1-Gluc and PR8-PB2-Gluc respectively acquire a modified segment NS or PB2 of altered sizes, we speculated that the vRNA packaging might be affected.

**Figure 2:**
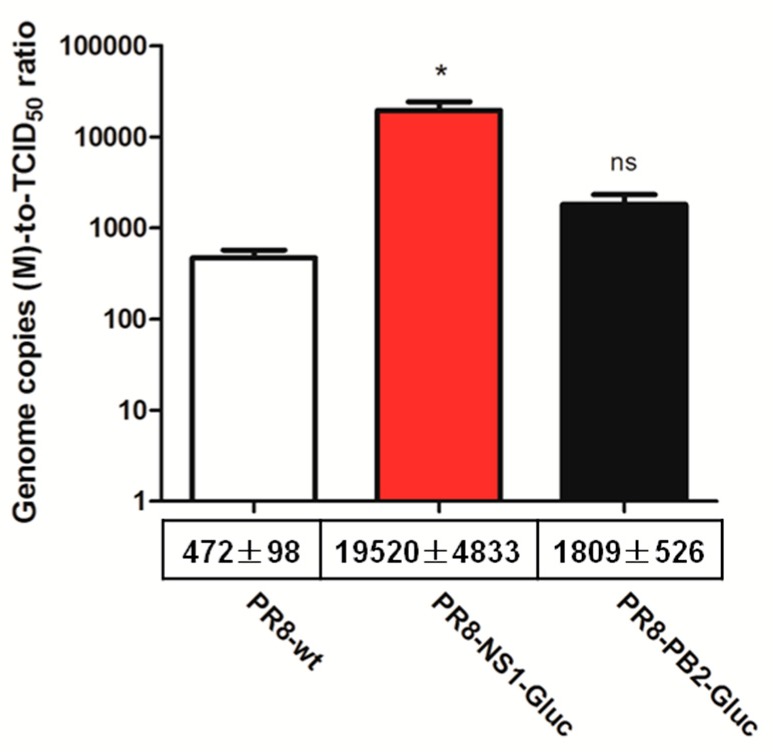
Relative genome copies-to-TCID_50_ ratio of reporter IAVs PR8-NS1-Gluc and PR8-PB2-Gluc. Indicated viruses derived from MDCK were titrated and extracted for vRNAs followed by quantification of segment M by qPCR. The genome copies-to-TCID50 ratio were evaluated. The standard deviations were calculated based on three replicates. * *p* < 0.05; ns, no significance, students’ *t* test.

To address this, the ratios of genome copies to the number of infectious unit (genome copies-to-TCID_50_ ratio) of parental PR8, PR8-NS1-Gluc and PR8-PB2-Gluc were determined. The number of copies of segment M was chosen to represent genome copies since vRNP M plays pivotal role during packaging, and it is the shortest segment besides NS [30]. We assumed that incorporation of vRNA M was less likely to be affected by modification of either PB2 or NS. As a result, the genome copies-to-TCID_50_ ratio of PR8-NS1-Gluc was drastically higher (41.4-fold, *p* < 0.05) compared to that of parental PR8, while that of PR8-PB2-Gluc was less affected, with a 3.8-fold increase (*p* > 0.05; Figure 2). These results suggest that PR8-NS1-Gluc produces ~41 folds more of the genome (M)-containing noninfectious particles for every such particle produced by the parental PR8 virus.

### 3.3. PR8-NS1-Gluc Produces Large Amounts of NS-Null Noninfectious Particles

**Figure 3:**
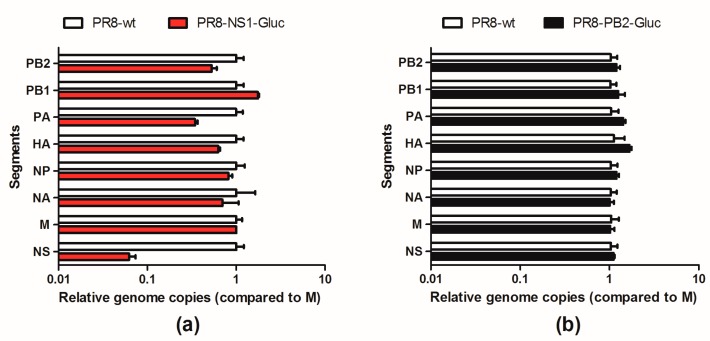
vRNA compositions of two reporter IAVs. MDCK derived PR8-NS1-Gluc (**a**) or PR8-PB2-Gluc (**b**) were extracted for vRNAs. The composition of vRNAs were examined by qPCR, and presented as the ratios of each vRNA segments versus M. The standard deviations were calculated based on three replicates.

In order to investigate whether the altered genome copies-to-TCID_50_ ratio of reporter IAVs was caused by impaired packaging efficacy of vRNAs NS-Gluc or PB2-Gluc, respectively, the vRNA compositions of PR8-NS1-Gluc and PR8-PB2-Gluc were examined. MDCK-derived virus stocks were extracted for vRNAs and relative amounts of eight segments were quantified by qPCR analysis. As shown in Figure 3a, the packaged vRNA NS-Gluc was drastically reduced compared to seven other segments. The NS-to-M ratio decreased for about 16-fold compared to that of PR8, suggesting large amounts of NS-null noninfectious particles were produced among PR8-NS1-Gluc virions.

In contrast, the vRNA composition in virions of PR8-PB2-Gluc was not significantly affected, and the ratio of the eight segments was almost equal to that of parental PR8 (Figure 3b), which is in accordance with our finding that genome copies-to-TCID50 ratio of PR8-PB2-Gluc is not significantly affected (Figure 2).

### 3.4. The vRNA NS-Gluc is Less Competitive in Replication

**Figure 4:**
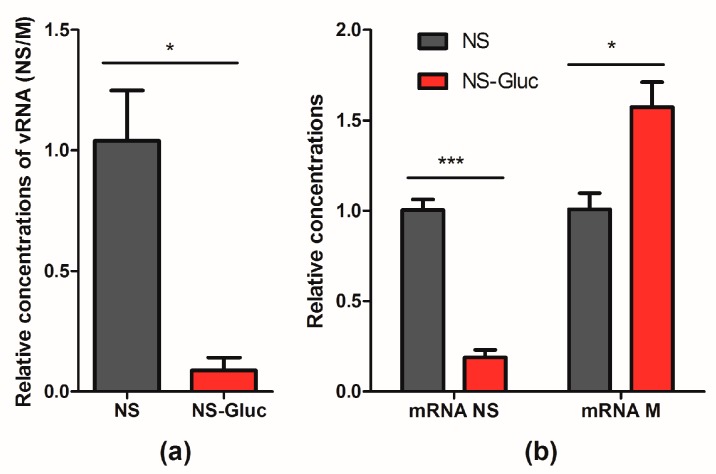
vRNA NS-Gluc is less competitive than natural NS in replication. RdRp assays were performed by cotransfecting pPolI-NS and pPolI-NS-Gluc respectively with pDZ-M as well as expression plasmids PA, PB1, PB2 and NP. At 30 h post transfection, the cells were harvested and total RNA were extracted. (**a**) The concentrations of vRNA NS and NS-Gluc were normalized to vRNA M, and the relative concentrations were presented; (**b**) The concentrations of mRNA NS, NS-Gluc and M were normalized to mRNA NP, and the relative concentrations were shown. The standard deviations were calculated based on three replicates. *** *p* < 0.001; * *p* < 0.05; students’ *t* test.

It is well known that the replication and transcription of the 8 segments are balanced in IAV by competing with each other for available polymerases. The competition can be affected by segment length, coding region, and UTRs [31]. Thus we asked whether the insertion of reporter Gluc negatively affected the competitive capacity of NS segment, which might cause the deficient incorporation. To address this, the replication efficacy of vRNA NS and NS-Gluc were respectively examined in the presence of natural vRNA M. Cell-based IAV RdRp assay was performed, and pPolI-NS and pPolI-NS-Gluc respectively were co-transfected with pDZ-M as well as plasmids expressing NP, PB1, PB2 and PA. As shown in Figure 4a, the relative replication of vRNA NS-Gluc was reduced by about 10-fold, suggesting that vRNA NS-Gluc is less competitive in replication than the parental NS.

The mRNA levels of NS and M were also examined. The mRNA NP, which is thought to be transcribed constantly under the control of promoter CMV, was set as an internal reference. The mRNA level of NS-Gluc was significantly decreased when compared to the parental NS (Figure 4b). Interestingly, we observed obvious increase of mRNA M in the presence of vRNA NS-Gluc compared to that in the presence of the parental vRNA NS (Figure 4b), which suggests that NS-Gluc is less competitive.

### 3.5. The Balance of the 8 Segments of Reporter IAVs Is Impaired

**Figure 5:**
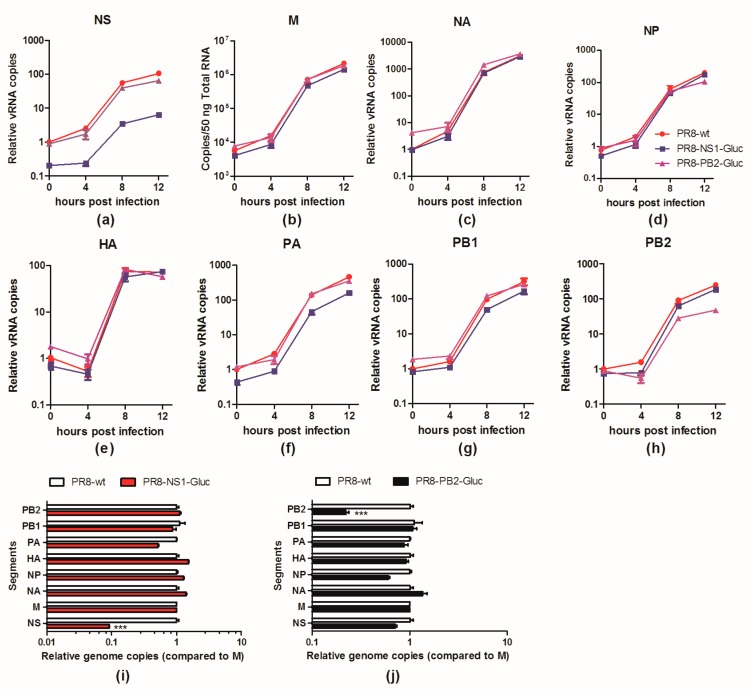
The replication of vRNA NS-Gluc and PB2-Gluc were impaired in infected cells. (**a**–**h**) Replication kinetics of indicated vRNAs in MDCK cells infected with PR8, PR8-NS1-Gluc, PR8-PB2-Gluc, respectively; (**i**,**j**) The relative concentrations of vRNAs in MDCK cells infected with indicated reporter IAVs were presented as the ratios of each vRNA segment versus M. The standard deviations were calculated based on three replicates. *** *p* < 0.001, students’ *t* test.

To further determine whether the less competitive replication of vRNA NS-Gluc is the cause for the impaired packaging of vRNA, the replication kinetics of vRNA segments in infected cells were examined. One cycle infection was performed by infecting MDCK cells with an MOI of 1 of parental PR8, PR8-NS1-Gluc or PR8-PB2-Gluc, respectively, and the relative amounts of vRNAs in infected cells were monitored. As shown in Figure 5, the replication of vRNA NS-Gluc was impaired by about 1 log all through the infection (Figure 5a), while none of the other seven segments were obviously affected except for PA, which was slightly reduced by 2–3 fold at different phases (Figure 5b–h). Interestingly, we also observed a slight reduction of vRNA PB2-Gluc replication in PR8-PB2-Gluc infected cells (Figure 5h). The impaired replication greatly reduced the amounts of vRNAs NS-Gluc or PB2-Gluc available for packaging (Figure 5i,j).

These results clearly demonstrate that modification of vRNA segments alters their replication property in infected cells. The reduced amount of vRNA NS-Gluc may predominately contribute to the NS-null noninfectious particles.

## 4. Discussion

Reporter viruses provide a powerful tool to study infections; however, their development and application usually encounter huge challenges. First, introduction of foreign genes or modification of existing viral genes might alter the virological properties, such as delay of replication kinetics or reduced virulence. Second, such alterations can create an evolutionary pressure, leading to the loss of reporter genes [32]. For example, a recombinant bunyavirus expressing GFP fused to the N-terminus of Gc is highly attenuated, which might be due to the fact that GFP directly inhibited the function of Gc, while replacing GFP with mCherry partially solved the problem [33]. As another example, expression of Renilla luciferase attenuated respiratory syncytial virus (RSV) in vivo, but expression of Katushka2 did not, suggesting that the observed attenuation was purely caused by the reporter protein itself rather than the changes to the virus genome [34]. However, for most reporter viruses, how reporter expression can cause attenuation remains unclear. It’s more likely to be through multiple mechanisms rather than by a single one. This study investigated the mechanisms underlying attenuation of two reporter IAVs, PR8-NS1-Gluc and PR8-PB2-Gluc [24,29]. Our data clearly demonstrated that elongation of either vRNA segment NS or PB2 reduced their replication efficacy respectively (Figure 4 and Figure 5). Subsequently, the incorporation of NS-Gluc into progeny virions was impaired, producing a large amount of NS-null noninfectious particles. Although we didn’t observe equivalent reduction of packaged vRNA PB2-Gluc when compared to other segments, this might due to the critical role of vRNP PB2 during the genome-packaging process [30].

Due to the segmented nature, replication of IAV genome is much more complicated. It has been reported that IAV balances the replication and transcription of its multiple genome segments by competition between them. The competition could be affected by segment length, coding region, and UTRs, of which any change might result in balance disruption [31]. It could be hypothesized that the impaired competition property of one segment can cause reduced replication and transcription, subsequently interfering with the balance of the genome segments. In the case of PR8-NS1-Gluc, the replication of vRNA NS-Gluc showed a drastic reduction (>1 log compared to M). Although in the case of PR8-PB2-Gluc, the reduction in replication of vRNA PB2-Gluc was less drastic (about 5 fold), this is reasonable, since the sequence changes relative to the length of the segment are much more important in the NS-segment as compared to those in the PB2 segment. However, since the ratio of the eight segments vRNAs is quite dynamic in the infection (Supplementary Figure S1), caution should be taken in analyzing and interpreting the kinetics of vRNA replication of recombinant reporter IAVs using the parental PR8 as a control.

The packaging of IAV genome segments into progeny virions is also a very complicated process. Previous studies address a selective model in which a complete set of eight vRNA segments is selectively packaged into each progeny virion [35,36,37]. It was proposed that the IAV segments were usually packaged into virions as an interlinked complex of 8 vRNPs, however, the contribution of each vRNA segment to virion production is different. The vRNPs PB2, PA, NP and M play a more pivotal role during the packaging process than NS and others [30]. This may explain why we observed large amounts of NS-null noninfectious particles, but no PB2-null ones.

As we proposed that the mechanism underlying attenuation of reporter viruses is comprehensive, we could not exclude the possibility that elongation of IAV segments directly interfere with the packaging efficacy due to altered sizes. Also, the reduced replication of NS or PB2 may decrease the expression of NS1 or PB2 proteins. Therefore, the NS1-mediated evasion of cellular innate immune response and PB2 related RdRp activity might also be affected [38,39], resulting in impairment of virus growth.

The loss of competition of elongated segment in replication and transcription could be restored. Providing more RdRp’s or introducing mutations that enhance the activity of RdRp’s could alleviate the competition between segments [28,31], while stabilizing the pan-handle structure of a vRNA segment can increase its binding to RdRp’s, promoting the competitiveness [31]. Based on our finding that impaired replication of corresponding vRNAs of modified segments fundamentally contribute to the attenuation, we propose that introduction of extra mutations that could compensate for the impairment might restore the viral infectivity and virulence.

In summary, this study unravels a mechanism underlying attenuation of two reporter IAVs, and provide feasible directions for their further optimization.

## Figures and Tables

**Table 1:** Segment specific primers used for qPCR analysis.

Segments	Forward Primer	Reverse Primer
PB2	5’-GTGCCTTCATCTGGGTCT -3’	5’-TTGGGACATTTGATACCG -3’
PB1	5’-AACTGGAGCACCGCAACT-3’	5’-AAGGAAAGCCATCGCCTC-3’
PA	5’-GATTCCTTTCGTCAGTCC-3’	5’-TCTAAGTGGTCGTGGTGT-3’
HA	5’-GAACTATTACTGGACCTTGC-3’	5’-CCTATTGTGACTGGGTGTAT-3’
NP	5’-GTATGGACCTGCCGTAGC-3’	5’-CTCTTGGGAGCACCTTCG-3’
NA	5’-AAACGGAGTAAAGGGATT-3’	5’-CAGGATGTTGAACGAAAC-3’
M	5’-CTTCTAACCGAGGTCGAAAC-3’	5’-CGTCTACGCTGCAGTCCTC-3’
NS	5’-ATTTCACCATTGCCTTCT-3’	5’-GGTCTCCCATTCTCATTAC-3’

## Supplementary Materials

The following are available online at http://www.mdpi.com/1999-4915/10/12/679/s1, Figure S1: The balance of the eight segments vRNAs is dynamic during the infection of IAV PR8 infection.

## References

[1] WHO (2018). 2018 Influenza (Seasonal) Fact Sheet. http://www.who.int/mediacentre/factsheets/fs211/en/.

[2] Resa-Infante P., Jorba N., Coloma R., Ortin J. (2011). The influenza virus RNA synthesis machine: Advances in its structure and function. RNA Biol..

[3] Eisfeld A.J., Neumann G., Kawaoka Y. (2015). At the centre: Influenza A virus ribonucleoproteins. Nat. Rev. Microbiol..

[4] Neumann G., Brownlee G.G., Fodor E., Kawaoka Y. (2004). Orthomyxovirus replication, transcription, and polyadenylation. Curr. Top. Microbiol. Immunol..

[5] Desselberger U., Racaniello V.R., Zazra J.J., Palese P. (1980). The 3′ and 5′-terminal sequences of influenza A, B and C virus RNA segments are highly conserved and show partial inverted complementarity. Gene.

[6] Fodor E., Pritlove D.C., Brownlee G.G. (1994). The influenza virus panhandle is involved in the initiation of transcription. J. Virol..

[7] Ferhadian D., Contrant M., Printz-Schweigert A., Smyth R.P., Paillart J.C., Marquet R. (2018). Structural and Functional Motifs in Influenza Virus RNAs. Front. Microbiol..

[8] Perez D.R., Angel M., Gonzalez-Reiche A.S., Santos J., Obadan A., Martinez-Sobrido L. (2017). Plasmid-Based Reverse Genetics of Influenza A Virus. Methods Mol. Biol..

[9] Neumann G., Watanabe T., Ito H., Watanabe S., Goto H., Gao P., Hughes M., Perez D.R., Donis R., Hoffmann E. (1999). Generation of influenza A viruses entirely from cloned cDNAs. Proc. Natl. Acad. Sci. USA.

[10] Isel C., Munier S., Naffakh N. (2016). Experimental Approaches to Study Genome Packaging of Influenza A Viruses. Viruses.

[11] Marsh G.A., Hatami R., Palese P. (2007). Specific residues of the influenza A virus hemagglutinin viral RNA are important for efficient packaging into budding virions. J. Virol..

[12] Basler C.F., Aguilar P.V. (2008). Progress in identifying virulence determinants of the 1918 H1N1 and the Southeast Asian H5N1 influenza A viruses. Antiv. Res..

[13] Pica N., Langlois R.A., Krammer F., Margine I., Palese P. (2012). NS1-truncated live attenuated virus vaccine provides robust protection to aged mice from viral challenge. J. Virol..

[14] Mueller S., Coleman J.R., Papamichail D., Ward C.B., Nimnual A., Futcher B., Skiena S., Wimmer E. (2010). Live attenuated influenza virus vaccines by computer-aided rational design. Nat. Biotechnol..

[15] Yang C., Skiena S., Futcher B., Mueller S., Wimmer E. (2013). Deliberate reduction of hemagglutinin and neuraminidase expression of influenza virus leads to an ultraprotective live vaccine in mice. Proc. Natl. Acad. Sci. USA.

[16] Pena L., Sutton T., Chockalingam A., Kumar S., Angel M., Shao H., Chen H., Li W., Perez D.R. (2013). Influenza viruses with rearranged genomes as live-attenuated vaccines. J. Virol..

[17] Nogales A., DeDiego M.L., Topham D.J., Martinez-Sobrido L. (2016). Rearrangement of Influenza Virus Spliced Segments for the Development of Live-Attenuated Vaccines. J. Virol..

[18] Hai R., Garcia-Sastre A., Swayne D.E., Palese P. (2011). A Reassortment-Incompetent Live Attenuated Influenza Virus Vaccine for Protection against Pandemic Virus Strains. J. Virol..

[19] Hai R., Krammer F., Tan G.S., Pica N., Eggink D., Maamary J., Margine I., Albrecht R.A., Palese P. (2012). Influenza viruses expressing chimeric hemagglutinins: Globular head and stalk domains derived from different subtypes. J. Virol..

[20] Breen M., Nogales A., Baker S.F., Martinez-Sobrido L. (2016). Replication-Competent Influenza A Viruses Expressing Reporter Genes. Viruses.

[21] Fukuyama S., Katsura H., Zhao D., Ozawa M., Ando T., Shoemaker J.E., Ishikawa I., Yamada S., Neumann G., Watanabe S. (2015). Multi-spectral fluorescent reporter influenza viruses (Color-flu) as powerful tools for in vivo studies. Nat. Commun..

[22] Lo M.K., Nichol S.T., Spiropoulou C.F. (2014). Evaluation of luciferase and GFP-expressing Nipah viruses for rapid quantitative antiviral screening. Antiv. Res..

[23] Czako R., Vogel L., Lamirande E.W., Bock K.W., Moore I.N., Ellebedy A.H., Ahmed R., Mehle A., Subbarao K. (2017). In Vivo Imaging of Influenza Virus Infection in Immunized Mice. mBio.

[24] Heaton N.S., Leyva-Grado V.H., Tan G.S., Eggink D., Hai R., Palese P. (2013). In vivo bioluminescent imaging of influenza a virus infection and characterization of novel cross-protective monoclonal antibodies. J. Virol..

[25] Manicassamy B., Manicassamy S., Belicha-Villanueva A., Pisanelli G., Pulendran B., Garcia-Sastre A. (2010). Analysis of in vivo dynamics of influenza virus infection in mice using a GFP reporter virus. Proc. Natl. Acad. Sci. USA.

[26] Tran V., Moser L.A., Poole D.S., Mehle A. (2013). Highly sensitive real-time in vivo imaging of an influenza reporter virus reveals dynamics of replication and spread. J. Virol..

[27] Reuther P., Gopfert K., Dudek A.H., Heiner M., Herold S., Schwemmle M. (2015). Generation of a variety of stable Influenza A reporter viruses by genetic engineering of the NS gene segment. Sci. Rep..

[28] Cai H., Liu M., Russell C.J. (2018). Directed evolution of an influenza reporter virus to restore replication and virulence and enhance non-invasive bioluminescence imaging in mice. J. Virol..

[29] Li P., Cui Q., Wang L., Zhao X., Zhang Y., Manicassamy B., Yang Y., Rong L., Du R. (2018). A Simple and Robust Approach for Evaluation of Antivirals Using a Recombinant Influenza Virus Expressing Gaussia Luciferase. Viruses.

[30] Gao Q., Chou Y.Y., Doganay S., Vafabakhsh R., Ha T., Palese P. (2012). The influenza A virus PB2, PA, NP, and M segments play a pivotal role during genome packaging. J. Virol..

[31] Widjaja I., de Vries E., Rottier P.J., de Haan C.A. (2012). Competition between influenza A virus genome segments. PLoS ONE.

[32] Falzarano D., Groseth A., Hoenen T. (2014). Development and application of reporter-expressing mononegaviruses: Current challenges and perspectives. Antiv. Res..

[33] Shi X., van Mierlo J.T., French A., Elliott R.M. (2010). Visualizing the replication cycle of bunyamwera orthobunyavirus expressing fluorescent protein-tagged Gc glycoprotein. J. Virol..

[34] Hotard A.L., Shaikh F.Y., Lee S., Yan D., Teng M.N., Plemper R.K., Crowe J.E., Moore M.L. (2012). A stabilized respiratory syncytial virus reverse genetics system amenable to recombination-mediated mutagenesis. Virology.

[35] Fujii Y., Goto H., Watanabe T., Yoshida T., Kawaoka Y. (2003). Selective incorporation of influenza virus RNA segments into virions. Proc. Natl. Acad. Sci. USA.

[36] Noda T., Kawaoka Y. (2010). Structure of influenza virus ribonucleoprotein complexes and their packaging into virions. Rev. Med. Virol..

[37] Noda T., Sugita Y., Aoyama K., Hirase A., Kawakami E., Miyazawa A., Sagara H., Kawaoka Y. (2012). Three-dimensional analysis of ribonucleoprotein complexes in influenza A virus. Nat. Commun..

[38] Fernandez-Sesma A., Marukian S., Ebersole B.J., Kaminski D., Park M.S., Yuen T., Sealfon S.C., Garcia-Sastre A., Moran T.M. (2006). Influenza virus evades innate and adaptive immunity via the NS1 protein. J. Virol..

[39] Fodor E. (2013). The RNA polymerase of influenza A virus: Mechanisms of viral transcription and replication. Acta Virol..

