# Supplementary material for: A Mechanism Underlying Attenuation of Recombinant Influenza A Viruses Carrying Reporter Genes
Source: Viruses. 2018 Nov 30;10(12):679. doi: 10.3390/v10120679 (PMC6316390; doi:10.3390/v10120679)
Supplement: Supplementary file 1 [file viruses-10-00679-s001.pdf]

viruses

MDPI

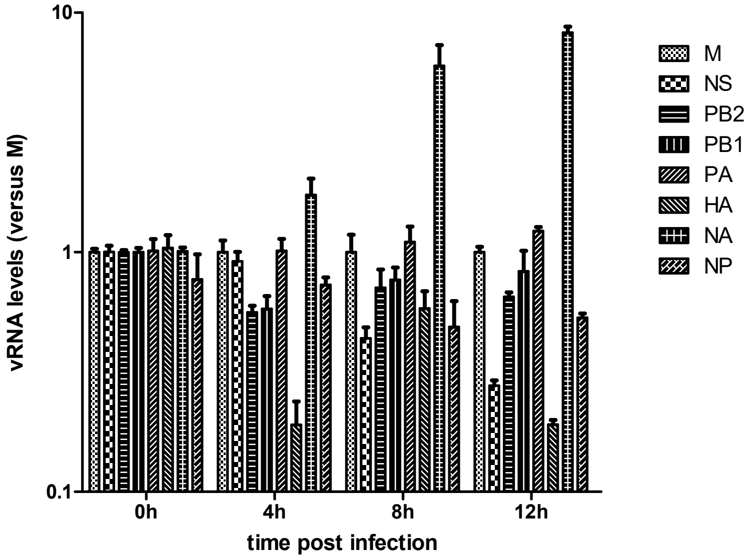

Figure S1. The balance of the eight segments vRNAs is dynamic during the infection of IAV PR8 infection. MDCK cells were infected with IAV PR8 at an MOI of 1 $TCID_{50}$/cell. At indicated time points, the cells were harvested and total RNAs were extracted for reverse transcription using a universal 3′ primer 5′-GATCGCTCTTCTGGGAGCRAAAGCAGG-3′ and qPCR analysis with segment specific primers separately. The relative concentrations of vRNAs were normalized to that of Time 0h and presented as the ratios of each vRNA segments versus M. The standard deviations were calculated based on three replicates.
